# A Florida-Based Analysis of Bowel Surgery Outcomes: Does Income Predict Recovery?

**DOI:** 10.7759/cureus.111764

**Published:** 2026-06-29

**Authors:** Heather Lumley, Benjamin S Crooks, Suzanne I Riskin, McHenry Mauger

**Affiliations:** 1 General Surgery, Keesler Air Force Base, Biloxi, USA; 2 Foundational Sciences, Nova Southeastern University Dr. Kiran C. Patel College of Osteopathic Medicine, Clearwater, USA; 3 Biostatistics, Nova Southeastern University Dr. Kiran C. Patel College of Allopathic Medicine, Davie, USA

**Keywords:** bowel repair, bowel surgery, colorectal, florida, general surgery, length of stay, median income, operative, readmission, socioeconomic status

## Abstract

Major small and large bowel surgeries are commonly performed in the United States (U.S.) for conditions that are associated with colorectal cancer, inflammatory bowel disease, and bowel obstruction. Factors such as hospital length of stay (LOS) and postoperative readmission rates are among the many outcome measures that could serve as markers for evaluating surgical effectiveness and the risk of complications. Additionally, socioeconomic status (SES) has been widely used as a predictor of postoperative outcomes across medical disciplines. Meanwhile, the relationship between hospital LOS, readmission rates, and SES has yet to be fully elucidated. As such, this study aims to evaluate whether county median household income, as a measure of SES in Florida, correlates with hospital LOS and 15-day potentially preventable readmission rates following major bowel procedures. Publicly available data on readmission rates and LOS were analyzed for major small- and large-bowel procedures, but data were not available for all counties in the state. This analysis included 48 of the 67 counties in the state. Data on postoperative outcomes were compared with the county median household income, which was stratified into quintiles. The second, third, and fourth quintile ranges included all of Florida counties. The vast majority (98.5%) of these counties were in the second and third quintiles. Regression analysis demonstrated no significant relationship between county median income and LOS. Similarly, comparisons of median income across the three readmission categories showed no statistically significant differences in the Kruskal-Wallis test. Despite demonstrating a limited association, these findings give valuable insight into the complexity of SES and postoperative outcomes, offering a clearer framework for understanding how socioeconomic conditions intersect with clinical factors. Additionally, median annual household income alone may not adequately capture the socioeconomic and structural factors influencing surgical recovery. Future research should prioritize obtaining complete readmission datasets, incorporate additional SES markers such as education, insurance status, and healthcare access, and conduct analyses at the patient- or neighborhood-level to provide a more refined view of the correlations. More granular evaluation may clarify if and which socioeconomic factors most strongly influence postoperative outcomes and guide targeted interventions to improve equity in surgical care.

## Introduction

Major small and large bowel surgeries are commonly performed in the United States (U.S.) for conditions that are associated with colorectal cancer, inflammatory bowel disease, and bowel obstruction, with over half a million surgeries being performed annually. Specifically, small and large bowel procedures are relatively common in the U.S., with over 300,000 small bowel obstruction surgeries and over 600,000 colon surgeries performed annually [1]. These procedures often include resection or removal of diseased areas of the bowel or widening of narrow passages [2]. Operations for resections of the small and large bowel can be done openly or laparoscopically. Open surgery may be more beneficial for complex cases or those involving multiple areas or organs. In contrast, laparoscopic surgery can be associated with lower postoperative pain, shorter hospital stays, and a quicker return to usual activities [3].

Length of stay (LOS) after surgery is an important indicator of hospital efficiency, outcomes, and risk of adverse events or complications. A shorter LOS commonly suggests quicker recovery and better patient outcomes, while a prolonged LOS may indicate complications or inefficiencies in care. In the U.S., the average hospital LOS after an open bowel resection is approximately five to seven days. In contrast, a minimally invasive approach or laparoscopic surgery can shorten it to approximately three to five days [4]. Common complications that can extend the LOS include surgical site infection, intra-abdominal abscess, anastomotic leakage, fistulization, ileus, and bleeding [5]. There is no specific average length of bowel resection during surgery, as it depends on each patient's needs and the nature of the specific condition being treated. Surgeons resect the minimum necessary to avoid resection of more than half of the small bowel, as more than this can lead to short bowel syndrome, which can cause issues with nutrient absorption [6].

An important factor in determining the proficiency of hospital teams and helping ensure quality patient care with minimal complications is the 15-day readmission rate after initial discharge following surgery. The readmission rate can be described as the potentially preventable readmission rate (PPR), indicating the patient would not have required readmission if they had received first-rate care during their initial hospital stay and proper coordination with discharge planning for the outpatient setting [7]. The average PPR in Florida is approximately 11% across age groups and illness severity [7]. Readmissions can be expensive, difficult to manage, and indicative of inefficiencies in the recovery process, potentially requiring a higher standard of post-surgery management within a hospital [8].

Socioeconomic status (SES) can influence a variety of healthcare outcomes. The American Psychological Association defines SES as an individual’s or group’s relative position within a social hierarchy, typically measured through income, educational attainment, and occupational prestige [9]. Data have demonstrated that individuals with lower SES often face barriers such as limited access to healthcare facilities and lower-quality care. These patients are less likely to receive preventative care, leading to higher rates of chronic illness and higher overall morbidity and mortality [10]. Lower SES has also been shown to be associated with higher rates of complications, including abnormal heart rhythms, surgical wound infections, and heart attacks [11]. Median household income is a consistent indicator of economic well-being at the individual and community levels, making it one of the most effective indicators of SES. The median household income in the U.S. was $77,719 in 2023 and $77,839 in 2022 [12]. Florida ranks 31st among other states with a median household income of $73,311 in 2023 [12]. The goal of our study is to examine the correlation between SES (using household income as the marker) and postoperative outcomes, including LOS and 15-day PPR, following small- and large-bowel procedures.

## Materials and methods

The approach involved downloading publicly available data from Florida Health Finder on major small- and large-bowel procedures performed between April 2022 and March 2023 [13]. The procedures were identified using the All Patient Refined Diagnosis-Related Groups codes 230 and 231, which correspond to all major small- and large-bowel procedures [7,14]. Any facility with fewer than 30 surgeries performed during the designated time frame did not have results displayed in the table and were not included in the analysis. The originally downloaded data was organized by city. We manually added the county designations for each listed hospital using cross-referenced zip codes from the Florida Department of Health. Of the 67 Florida counties, only 48 had eligible procedure data and were included in the analysis.

The collected data investigated both LOS and 15-day PPR as outcomes following bowel repair surgery. LOS data were calculated as each hospital’s average, and hospitals without LOS data were excluded from the analysis. We aggregated those averages across all hospitals in the county into a single value. This allowed comparison between the county LOS figure and the median income. Fifteen-day readmission rates were reported into one of four categories: fewer readmissions than expected given how sick patients were (better than expected), expected number of readmissions given how sick patients were (as expected), more readmissions than expected given how sick patients were (worse than expected), or not applicable (N/A) with not enough data to calculate [13]. These readmission categories were stratified into numerical groups: (1) worse than expected, (2) as expected, and (3) better than expected. Any hospitals without readmission data were not included in the analysis.

To compare this data with selected SES data for Florida based on the counties included, we retrieved the Social and Economic Factors Report (SEFR) from the Florida Health Charts Community Dashboard for each of the 48 counties with available data. The SEFR was downloaded for 2022 and 2023 for each included county to span the same time frame as the bowel procedure data and includes information from the U.S. Census Bureau American Community Survey five-year estimates [15]. Median household income was selected as the primary SES indicator for each county due to its consistent availability and use in public health research. For each county, an average of the 2022 and 2023 median household income data was calculated to represent income over the study period.

This median annual household income was then stratified into quintiles to indicate SES. Based on data from the 2023 U.S. Census, median household income can be divided into quintiles. The lowest quintile had incomes of $33,000 or less. The second quintile had incomes up to $62,200. Households in the third quintile had incomes up to $101,000, and those in the fourth quintile had incomes up to $165,300. The fifth quintile included households with incomes above $165,300 [16]. By categorizing households into these wealth quintiles, this approach enabled more detailed statistical analysis and comparison of postoperative healthcare outcomes across distinct segments of the large dataset.

Data analysis was performed using JMP® Pro 18.0.0 (SAS Institute Inc., Cary, NC, USA) and R 4.2.2 (R Foundation for Statistical Computing, Vienna, Austria), in collaboration with a biostatistician. Descriptive statistics, including standard deviation (SD), mean, median, minimum (min), and maximum (max), were calculated for each variable, LOS, and 15-day PPR, along with Pearson correlation coefficients. Additional analysis used a linear regression model to assess whether county median annual household income was a significant predictor of hospital LOS. Additionally, a nonparametric group comparison was conducted using the Kruskal-Wallis test to evaluate differences in median household income across the groups for 15-day PPR.

## Results

LOS data results

**Figure 1:**
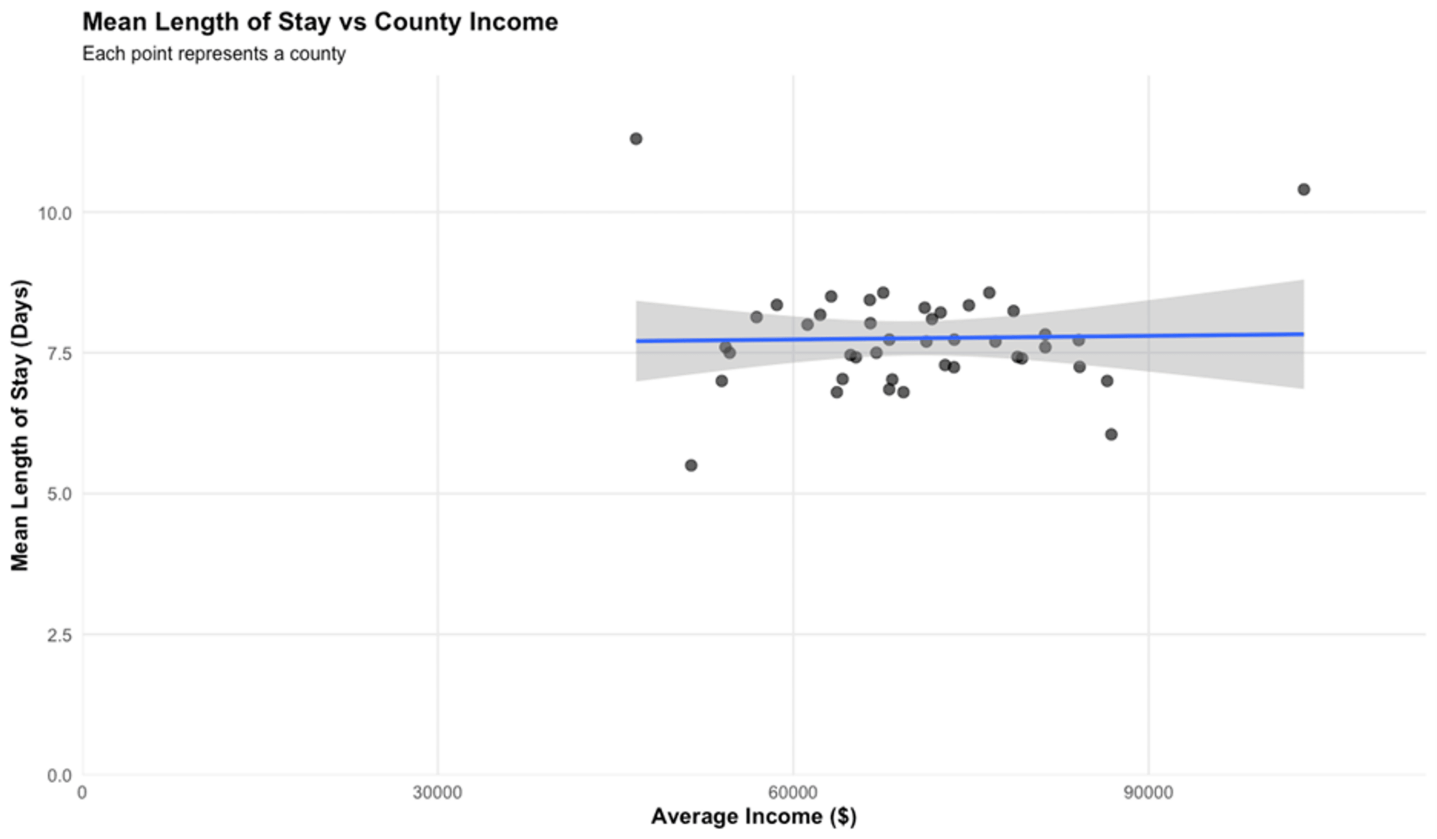
Linear regression graph (LOS by median annual household income) Simple linear regression of LOS by county median annual household income. Each point represents data from a single county. The blue line shows the regression comparison. LOS: length of stay

A simple linear regression analysis (Table 1) was used to determine the relationship between county median annual household income and average LOS for available county data in 2022 and 2023. A horizontal regression line is observed (as shown in Figure 1), indicating a zero regression coefficient, and the standard error for the median household income variable is less than 0.001. These findings indicate no statistically significant linear relationship between the two variables, suggesting that between 2022 and 2023, county median income did not affect average hospital LOS for patients recovering from bowel surgery. The results of a pairwise correlation between the LOS and median annual household income per county (Table 2) showed a 95% confidence interval (CI) of (-0.28, 0.32) and a Pearson correlation coefficient of 0.02, indicating a statistically insignificant, very weak positive association.

**Table 1:** Linear regression analysis (LOS by median annual household income) Data is presented as regression coefficient estimates with standard errors. Statistical significance was defined as p < 0.05. LOS: length of stay

Term	Estimate	Standard error	t value	Pr(>|t|)
Intercept	7.61	0.98	7.77	<0.001
Median income	<0.001	<0.001	0.16	0.877

**Table 2:** Pearson’s pairwise correlation coefficient (LOS vs. median annual household income) Data are presented as correlation coefficients with 95% CIs. Statistical significance is defined as p < 0.05. LOS: length of stay, CIs: confidence intervals

	Correlation coefficient	95% CI	p-value
LOS vs. median income	0.02	-0.28, 0.32	0.877

15-day PPR results

Median annual household income descriptive statistics are shown (Table 3) for each of the three defined 15-day readmission groups to give a preliminary picture of data distribution. These figures represent the mean, median, SD, min, and max of annual incomes. The sample sizes were based on the total number of hospitals in the 48 included counties. They were as follows: group 1 (n = 43) for worse-than-expected readmission rates, group 2 (n = 101) for readmission rates as expected, and group 3 (n = 45) for better-than-expected readmission rates. The mean income for group 1 (more readmissions) was $68,800 (SD $6,960). The mean income for group 2 (expected readmissions) was $70,400 (SD $8,850), which was slightly higher. At $70,700 (SD $6,020), group 3 (fewer readmissions) had the highest mean median income. Each of these readmission rates had averages in the third quintile, consistent with the overall averages across Florida counties.

**Table 3:** Summary statistics (median annual household income vs. 15-day readmission group) Data are presented as mean annual income ± SD and median, with associated minimum (min) and maximum (max) values within each group. Group 1 has more readmissions than expected, group 2 has the expected number of readmissions, and group 3 has fewer readmissions than expected. In each group, the N value represents the total number of hospitals with reportable data across all Florida counties for that readmission group. Group comparison was performed using the Kruskal-Wallis test. Statistical significance is defined as p < 0.05. SD: standard deviation

Median income	Group 1 (more readmissions) (N = 43)	Group 2 (expected) (N = 101)	Group 3 (fewer readmissions) (N = 45)	p-value
Mean (SD)	68800 (6960)	70400 (8850)	70700 (6020)	0.702
Median (min, max)	68300 (46700, 81300)	68300 (46100, 103000)	71100 (56900, 84100)	-

**Figure 2:**
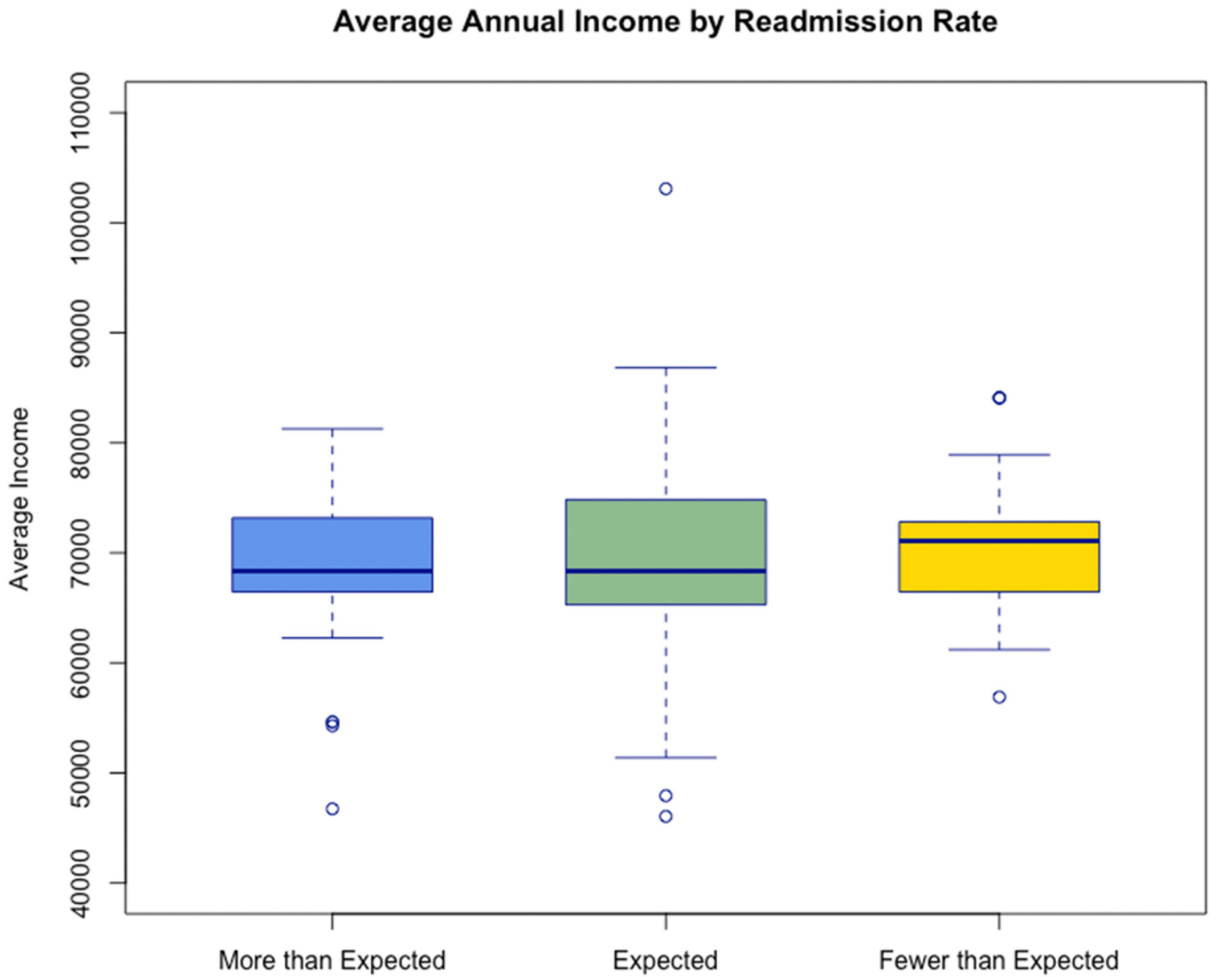
Boxplot (median annual household income vs. 15-day PPR group) Boxplot of median annual household income across the separate 15‑day PPR groups. Outliers are shown as points outside of the boxplot. PPR: preventable readmission rate

To formally evaluate the association between 15-day PPR groups and county median annual household income, a nonparametric group comparison was used (Table 3). For the three hospital 15-day readmission groups, the Kruskal-Wallis test was used to compare median income across the groups, yielding a p-value of 0.702. This finding suggests that whether a county hospital belongs to a group with more-than-expected, expected, or fewer-than-expected 15-day PPR does not appear to be significantly influenced by the county median annual household income. A visual representation of these 15-day PPR groups is shown in Figure 2, which presents the median annual household income for each group in a box plot, along with associated outliers.

Data availability by county

The 2023 U.S. Census was used to stratify annual median household income into quintiles based on the following income ranges: $0-33,000, $33,001-62,200, $62,201-101,000, $101,001-165,300, and $165,201 and above [16]. Table 4, which lists the counts and percentages of counties with and without available data, offers an essential summary of the availability of hospital readmission data across these different income quintiles. Since it directly measures the amount of missing data across all income levels, this information is essential for evaluating the external validity and potential biases of the study's conclusions. The observations in Table 4 indicate notable differences in data completeness. Only 14 counties (46.7%) had hospital data for the second income quintile (incomes up to $62,200), whereas the majority, 16 counties (53.3%), did not. Data completeness was much higher in the third income quintile (incomes up to $101,000), with 32 counties (88.9%) having accessible data and only four (11.1%) lacking it. For the fourth income quintile (incomes as high as $165,300), there was only one county in that quintile, and it reported hospital readmission data (100.0%). Notably, no data are available for the first and last quintiles. This is because no Florida counties fall within those annual median household income ranges. All 67 Florida counties fall within the second, third, and fourth quintile ranges.

**Table 4:** Counts and percentages of counties with or without readmission data by income quintile Data is presented in a table to show variability in data availability across counties. N represents the number of counties in each group, and the percentile (%) indicates the percentage of counties in each median-income quintile that have data available or unavailable for 67 Florida counties.

Income quintile	Hospital data available	Total counties within quintile	N counties with data (%)	N counties without data (%)
1: First	No	0	0	0
2: Second	Yes	30	14 (46.7%)	16 (53.3%)
3: Middle	Yes	36	33 (91.7%)	3 (8.3%)
4: Fourth	Yes	1	1 (100%)	0 (0%)
5: Last	No	0	0	0

## Discussion

The primary purpose of this study was to evaluate the correlation between SES, specifically county median household income, with LOS and 15-day PPR among surgeries performed on the small and large bowel. This differs from prior literature on the topic because no study has focused solely on county median income level as a measure of SES directly relating to bowel repair outcomes. Most prior studies examining SES and surgical outcomes have used multiple patient-level SES variables, encompassing personal, social, and economic factors, including income, insurance status, and education level [11,17]. Additionally, no prior study has focused exclusively on Florida using publicly available statewide hospital data, so we aimed to obtain a more refined, smaller-scale look at the data to appreciate differences that might be apparent within distinct communities. For our study, average LOS and 15-day PPR were the key variables evaluated at each hospital. However, because some data were unreported, only 48 of 67 Florida counties had reportable data included in our analysis to evaluate the association of median household incomes grouped into quintiles. As shown in Table 4 of the results section, counties with lower median annual household income (second quintile) had 53.3% of their hospitals' 15-day PPR data unreported. It is possible that if this data had been available, a more statistically significant relationship may have been detected. The full analysis of our study demonstrated a null hypothesis showing no statistically significant relationship between county median household income and average LOS or 15-day PPR.

Despite demonstrating a limited association, these findings provide important insight into the complexity of SES and postoperative outcomes. It shows that median annual income alone may not adequately capture the socioeconomic and structural factors influencing surgical recovery. It suggests that SES is more than just income and that the best way to measure it might be through a multidimensional composite rather than a single variable. While lower-income counties may lack sufficient resources, our analysis demonstrated that this does not contribute to less desirable surgical outcomes in LOS and readmissions. This understanding could provide more accurate guidance for evidence-based interventions and policy initiatives aimed at fostering health equity. Future research may further clarify whether median household income independently predicts postoperative outcomes. Studies should take a more comprehensive approach by including SES variables such as education level, access to healthcare, and defined minority groups. This should be done at the patient-to-patient or neighborhood level to avoid broad generalizations about entire counties and to avoid biases from individual neighborhoods that may influence data across the entire state of Florida. Applying these parameters should determine or narrow down which SES factors, if any, are statistically significant in affecting surgical outcomes for the large and small bowel.

Limitations

Our study evaluated SES as it relates to the median yearly household income of each county, leaving out other important variables that have been shown to impact LOS and 15-day PPR rates, including but not limited to social support, patient comorbidities, access to primary and post-acute care, hospital quality indicators, and the efficacy of discharge planning. This omission might obscure the true impact of income and result in an underspecified model by confounding or mediating the association with income. More detailed indicators, such as individual patient income or neighborhood-level data, may show different relationships, since the county median annual income may be too general a metric to capture the complex socioeconomic factors driving individual patient readmission outcomes. The data gathered on 15-day PPR, organized into three readmission-based groupings, may have affected the generalizability of our results. When a potentially continuous outcome variable is categorized, statistical power may be reduced, and important information about the extent of readmissions may be lost. Although classification can make analysis and interpretation easier, it frequently results in the loss of detail. A continuous measure may lose its intricate relationship to income when broken down into three general categories.

## Conclusions

The goal of our study was to determine the impact of SES, as measured by median annual household income, on the surgical outcomes of small- and large-bowel procedures, as measured by LOS and 15-day PPR. The analysis of county-level data in Florida between 2022 and 2023 revealed no statistically significant correlation between median annual household income and average hospital LOS or 15-day PPR. To improve future research on the relationship between socioeconomic characteristics and postoperative outcomes, the following recommendations are based on the observed limitations. Future research should place a high priority on obtaining complete and reliable 15-day readmission data for all counties, particularly those currently unreported in publicly available databases. Direct cooperation with hospital systems or state health departments, or the use of more extensive national archives, might be required for this. This is needed to confirm the current study's results, which show no association between median annual household income and postoperative outcomes. Additionally, future studies should investigate other socioeconomic factors beyond income, such as education, minority status, and access to healthcare. Based on our study's results, income levels alone may not be the best determinant of postoperative outcomes. Adjusting for these variables can enable future research to offer stronger, more generalizable understandings of the true effects of SES on hospital use and patient outcomes. This will ultimately guide focused interventions and health policy aimed at promoting health equity.

## Appendices

**Table 5:** Full breakdown of data availability by county

County	Median income quintile	# of listed hospitals	# of hospitals with included data	LOS data	Readmission data
Alachua	2nd	2	2	2 (100%)	2 (100%)
Baker	3rd	0	0	-	-
Bay	3rd	2	2	2 (100%)	2 (100%)
Bradford	2nd	0	0	-	-
Brevard	3rd	7	7	7 (100%)	7 (100%)
Broward	3rd	15	14	14 (93%)	15 (100%)
Calhoun	2nd	0	0	-	-
Charlotte	3rd	3	3	3 (100%)	3 (100%)
Citrus	2nd	2	2	2 (100%)	2 (100%)
Clay	3rd	2	2	2 (100%)	2 (100%)
Collier	3rd	4	4	4 (100%)	4 (100%)
Columbia	2nd	1	1	1 (100%)	1 (100%)
DeSoto	2nd	1	0	0 (0%)	1 (100%)
Dixie	2nd	0	0	-	-
Duval	3rd	10	9	9 (90%)	9 (90%)
Escambia	3rd	3	3	3 (100%)	3 (100%)
Flagler	3rd	1	1	1 (100%)	1 (100%)
Franklin	2nd	0	0	-	-
Gadsden	2nd	0	0	-	-
Gilchrist	2nd	0	0	-	-
Glades	2nd	0	0	-	-
Gulf	2nd	0	0	-	-
Hamilton	2nd	0	0	-	-
Hardee	2nd	0	0	-	-
Hendry	2nd	1	0	0 (0%)	0 (0%)
Hernando	2nd	3	1	1 (33%)	3 (100%)
Highlands	2nd	2	1	1 (50%)	2 (100%)
Hillsborough	3rd	11	11	11 (100%)	11(100%)
Holmes	2nd	1	0	0 (0%)	0 (0%)
Indian River	3rd	2	2	2 (100%)	2(100%)
Jackson	2nd	1	1	1 (100%)	1 (100%)
Jefferson	2nd	0	0	-	-
Lafayette	2nd	0	0	-	-
Lake	3rd	3	3	3 (100%)	3 (100%)
Lee	3rd	5	4	4 (80%)	5 (100%)
Leon	3rd	2	2	2 (100%)	2 (100%)
Levy	2nd	0	0	-	-
Liberty	2nd	0	0	-	-
Madison	2nd	0	0	-	-
Manatee	3rd	3	3	3 (100%)	3 (100%)
Marion	2nd	3	3	3 (100%)	3 (100%)
Martin	3rd	1	1	1 (100%)	1 (100%)
Miami-Dade	3rd	25	16	16 (64%)	21 (84%)
Monroe	3rd	2	1	1 (50%)	2 (100%)
Nassau	3rd	1	1	1 (100%)	1 (100%)
Okaloosa	3rd	3	3	3 (100%)	3 (100%)
Okeechobee	2nd	1	1	1 (100%)	1 (100%)
Orange	3rd	12	7	7 (58%)	11 (92%)
Osceola	3rd	5	4	4 (80%)	5 (100%)
Palm Beach	3rd	14	12	12 (86%)	13 (93%)
Pasco	3rd	6	5	5 (83%)	6 (100%)
Pinellas	3rd	12	8	8 (67%)	10 (83%)
Polk	3rd	5	4	4 (80%)	5 (100%)
Putnam	2nd	1	0	0 (0%)	1 (100%)
Santa Rosa	3rd	2	2	2 (100%)	2 (100%)
Sarasota	3rd	4	4	4 (100%)	4 (100%)
Seminole	3rd	4	4	4 (100%)	4 (100%)
St Johns	4th	1	1	1 (100%)	1 (100%)
St Lucie	3rd	3	3	3 (100%)	3 (100%)
Sumter	3rd	1	1	1 (100%)	1 (100%)
Suwannee	2nd	0	0	-	-
Taylor	2nd	1	0	0 (0%)	0 (0%)
Union	3rd	0	0	-	-
Volusia	3rd	6	5	5 (83%)	6 (100%)
Wakulla	3rd	0	0	-	-
Walton	3rd	1	1	1 (100%)	1 (100%)
Washington	2nd	1	0	0 (0%)	0 (0%)
